# Author Correction: Targeted scavenging of extracellular ROS relieves suppressive immunogenic cell death

**DOI:** 10.1038/s41467-021-24476-z

**Published:** 2021-07-01

**Authors:** Hongzhang Deng, Weijing Yang, Zijian Zhou, Rui Tian, Lisen Lin, Ying Ma, Jibin Song, Xiaoyuan Chen

**Affiliations:** 1 MOE key laboratory for analytical science of food safety and biology, College of Chemistry, Fuzhou University, Fuzhou, 350108 China; 2 Laboratory of Molecular Imaging and Nanomedicine (LOMIN), National Institute of Biomedical Imaging and Bioengineering (NIBIB), National Institutes of Health (NIH), Bethesda, MD 20892 USA

**Keywords:** Cancer therapy, Nanotechnology in cancer, Cancer therapy, Nanotechnology in cancer

Correction to: *Nature Communications* 10.1038/s41467-020-18745-6, published online 02 October 2020.

This Article contained an error in both the legend to Fig. 3 and the description of Fig. 3k in the Results section. The Fig. 3l legend incorrectly stated that the quantitative analysis was based on *n* = 5 independent replicates, this should have stated *n* = 3 independent replicates. In addition, the sentence in the Results section entitled ‘Investigation of PEG-TECM-NS/OLE-induced ICD and in vitro anticancer efficacy’ the following sentence ‘As shown in Fig. 3k, l, the percentage of late apoptotic cells (Annexin V-FITC and 7-AAD double stained) was 36.6% when treated with PEG-TECM-NS/OLE pretreated with pH 6.8.’ incorrectly stated that the percentage of late apoptotic cells was 36.6%; this sentence now reads ‘As shown in Fig. 3k, l, the percentage of late apoptotic cells (Annexin V-FITC and 7-AAD double stained) was 24.7% when treated with PEG-TECM-NS/OLE pretreated with pH 6.8.’ The pdf and HTML versions of the Article have been updated.

