# Supplementary material for: Targeted scavenging of extracellular ROS relieves suppressive immunogenic cell death
Source: Nat Commun. 2020 Oct 2;11:4951. doi: 10.1038/s41467-020-18745-6 (PMC7532538; doi:10.1038/s41467-020-18745-6)
Supplement: Supplementary file 1 — Supplementary Information [file 41467_2020_18745_MOESM1_ESM.pdf]

Supplementary Information

Targeted Scavenging of Extracellular ROS Relieves Suppressive Immunogenic Cell Death

Deng et. al.

Supplementary Methods

Materials and instruments. 3-mercaptopropionic acid, propylene sulfide, polyethylenimine (branched, Mw 10000), p-formylbenzoic acid, N,N'-Dicyclohexylcarbodiimide (DCC), 4-dimethylaminopyridine (DMAP) N,N,N’,N’-Tetramethyl-O-(1H-benzotriazol-1-yl)uronium hexafluorophosphate (HBTU), N,N-Diisopropylethylamine (DIEA), polyethylene glycol (PEG, Mn 8000) and 4-carboxybenzaldehyde were used as received from Sigma-Aldrich (Milwaukee, USA). Phosphate buffered saline (PBS), Dulbecco’s modified Eagle’s medium (DMEM), DNA fragmentation imaging kit (TUNEL) and Dead Cell Apoptosis Kit with Annexin V Alexa Fluor® 488 & 7 AAD were purchased from Thermo Fisher Scientific. All above materials were used as received. UV-vis absorption spectra were obtained on a Genesys 10S UV-Vis spectrophotometer (Thermo Scientific, Waltham, MA). Proton nuclear magnetic resonance (^{1}H-NMR) spectra were recorded on a Bruker AV300 scanner using D_2O, DMSO-d6 and $CDCl_3$ as the solvent. The size of nanoparticles were measured by a SZ-100 nano particles analyzer (HORIBA Scientific, USA). Transmission electron microscopy (TEM) images were acquired on a Tecnai TF30 transmission electron microscope (TEM) (FEI, Hillsboro, OR). The concentrations of zinc were detected by inductively coupled plasma optical emission spectroscopy (ICP-OES, Agilent 720-ES). Confocal microscopy images were acquired on a Zeiss LSM 780 microscope. X-ray photoelectron spectroscopic spectrum was tested with a high sensitivity Kratos AXIS 165 spectrometer. PA images were acquired with a Visual Sonic Vevo 2100 LAZR system. Flow cytometry analysis was performed on a BD Accuri C6 flowpps cytometry.

Animal model All animal experiments were performed under a National Institutes of Health Animal Care and Use Committee (NIHACUC) approved protocol. BALB/C mice (Harlan, Indianapolis, IN) were subcutaneously implanted with 1×10^6 of 4T1 cells.

Preparation of poly(propylene sulfide) (PPS). 3-meraptopropionic acid (0.5 mmol) was introduced in a schlenk tube under an inert atmosphere and was dissolved in THF. Sodium methylate (0.6 mmol, 0.5 M in methanol) was then added, and the mixture was stirred for 30 min at room temperature. Propylene sulfide (10 mmol) was added to the mixture and the mixture was stirred overnight at 60 ^{o}C. The solvent was removed, and the resulting viscous liquid was twice extracted with methanol.

Preparation of PEI-PPS. PEI-PPS was prepared *via* amidation reaction between PEI and PPS. Typically, to a solution of PEI (1.0 g 0.01 mmol) in 20 mL DMSO were added PPS(0.05 mol), HBTU (78 mg, 0.2 mmol), and DIEA (25.8 mg, 0.2 mmol). The solution was kept at room temperature under stirring for 24 h. The resulting mixture was dialyzed against water with 10 mmol GSH for 24 h and then freeze dried.

Preparation of CHO-PEG-CHO. Typically, to a solution of PEG (3.2 g, 0.4 mmol) in DCM (100 mL) were added p-formylbenzoic acid (0.6 g, 4 mmol), DCC (0.82 g, 4 mmol), and DMAP (0.12 g, 1 mmol). After being stirred for 24 h, the solution was filtered. The filtrate was concentrated, dissolved in isopropanol (20 mL), and cooled at 0 ^{o}C for 2 h. The resulting crystals were collected by filtration and washed with isopropanol and diethyl ether.

MTT Assays. In a 96 well plate, the cells (5×10^3 cells/well) were incubated with DMEM medium supplemented containing 10% fetal bovine serum (FBS), 1% L-glutamine, antibiotics penicillin (100 IU/mL), and streptomycin (100 μg/mL) for 24 h. A total of 20 μL subnatant solution of PEG-T^{ECM}-NS pretreated with pH 6.8 and 10 mM H_2O_2 after centrifugalizaiton in PBS was added to yield final nanoparticles concentrations. In an atomosphere with 5% CO_2, the cells were cultured at 37 ^{o}C for 24 h. 10 μL MTT (5 mg/mL) was added. After 4 h, aspirated the medium and added 100 μL DMSO. Absorbance of 590 nm of each well was measured using a microplate reader. The

relative cell viability (%) was determined by comparing the absorbance at 590 nm with control, which contained only culture medium. Data are presented as average ± SD (n=4).

Western blotting. 4T1 cells were washed using PBS and lysed using RIPA lysis buffer supplemented with complete protease inhibitor cocktail tablets. Twenty micrograms of proteins were separated by SDS-polyacrylamide gel electrophoresis. Then the protein were transferred onto polyvinylidene difluoride membranes. After blocking for 1 hour with 1.5% BSA, membranes were incubated with the primary antibody (anti-CRT, anti-HMGB1 and anti-β-actin-HRP) overnight. Antimouse or antirabbit antibodies conjugated with horseradish peroxidase were used as secondary antibody. Membranes were visualized with a chemiluminescent substrate. Anti-CRT, anti-HMGB1 and anti-β-actin-HRP were diluted according to the manufacturer's direction (1:1000). Antimouse and antirabbit antibodies conjugated with horseradish peroxidase were diluted according to the manufacturer's direction (1:1000).

Fluorescence immunostaining After different treatments, cells or tissues section samples were fixed by Z-fix solution. After blocking for 1 hour with 1.5% BSA, samples were incubated with the primary antibody (anti-CRT, anti-HMGB1 or anti-Collagen I) overnight. Antimouse or antirabbit antibodies conjugated with fluorescence were used as secondary antibody. Samples were visualized with a CLSM. Anti-CRT, anti-HMGB1 and anti-Collagen I were diluted according to the manufacturer's direction (1:500). Antimouse and antirabbit antibodies conjugated with fluorescence were diluted according to the manufacturer's direction (1:500).

Histomorphological analysis Tissues were examined for histopathology after staining with hematoxylin and eosin (H&E). Tumor, liver, spleen and kidney were collected and fixed for 24 h in 4% paraformaldehyde, then tissue samples were trimmed, embedded in paraffin, and cut into 5

μm thick sections for H&E assays according to the standard staining protocol. The photographs were taken using optical microscope (Leica DMI6000 B).

Supplementary Figures

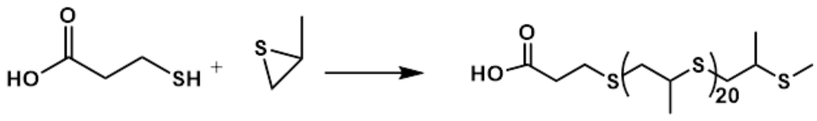

Supplementary Fig. 1 Synthesis route of PPS.

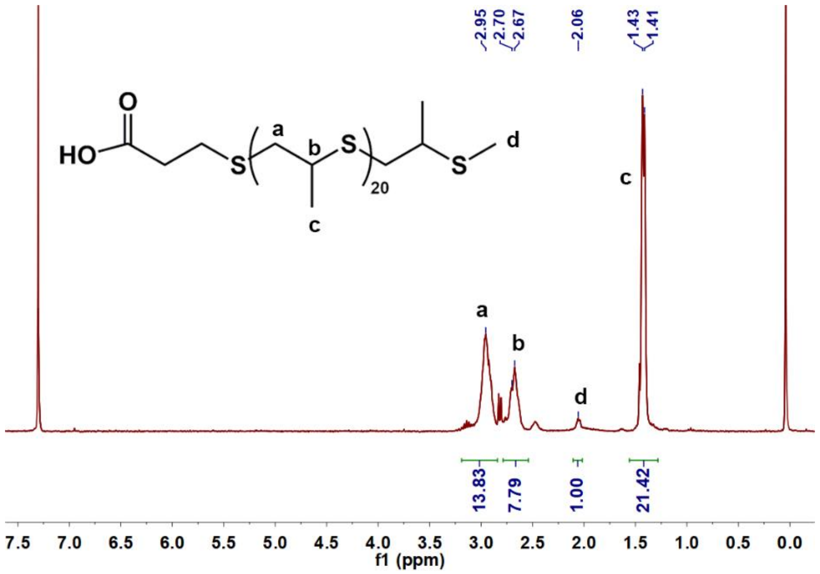

Supplementary Fig. 2 ^{1}H NMR of PPS. Repeated for three times in independent experiment.

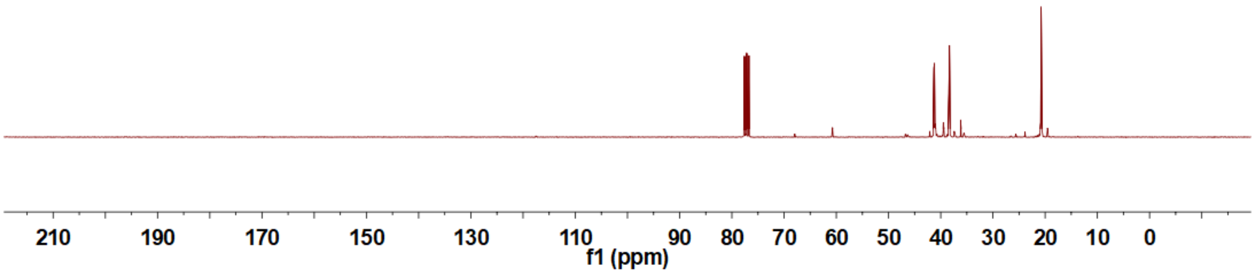

Supplementary Fig. 3 ^{13}C NMR of PPS. Repeated for three times in independent experiment.

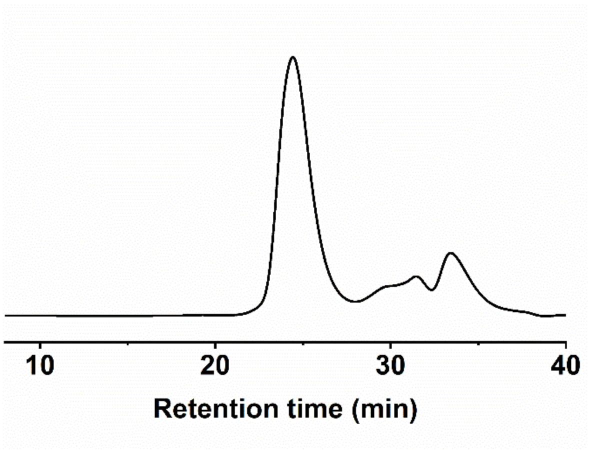

Supplementary Fig. 4 GPC result of PPS. Repeated for three times in independent experiment.

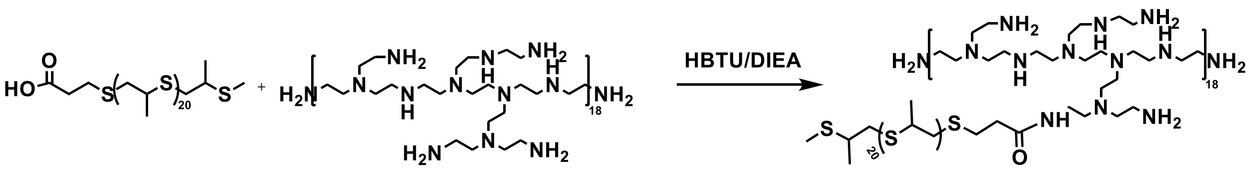

Supplementary Fig. 5 Synthesis route of PEI-PPS.

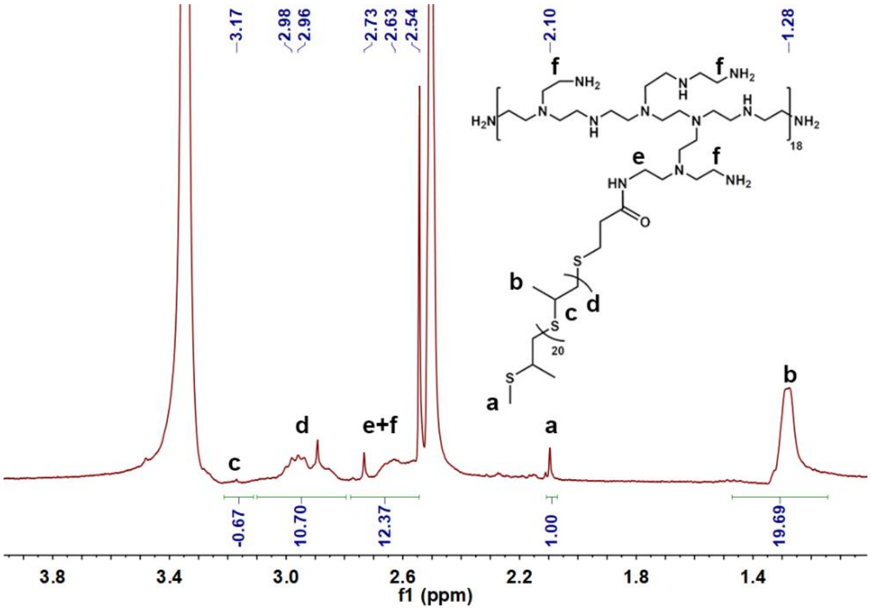

Supplementary Fig. 6 ^{1}H NMR of PEI-PPS. Repeated for three times in independent experiment.

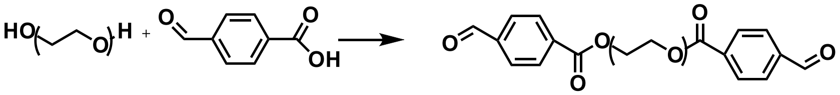

Supplementary Fig. 7 Synthesis route of dual aldehyde end groups of PEG.

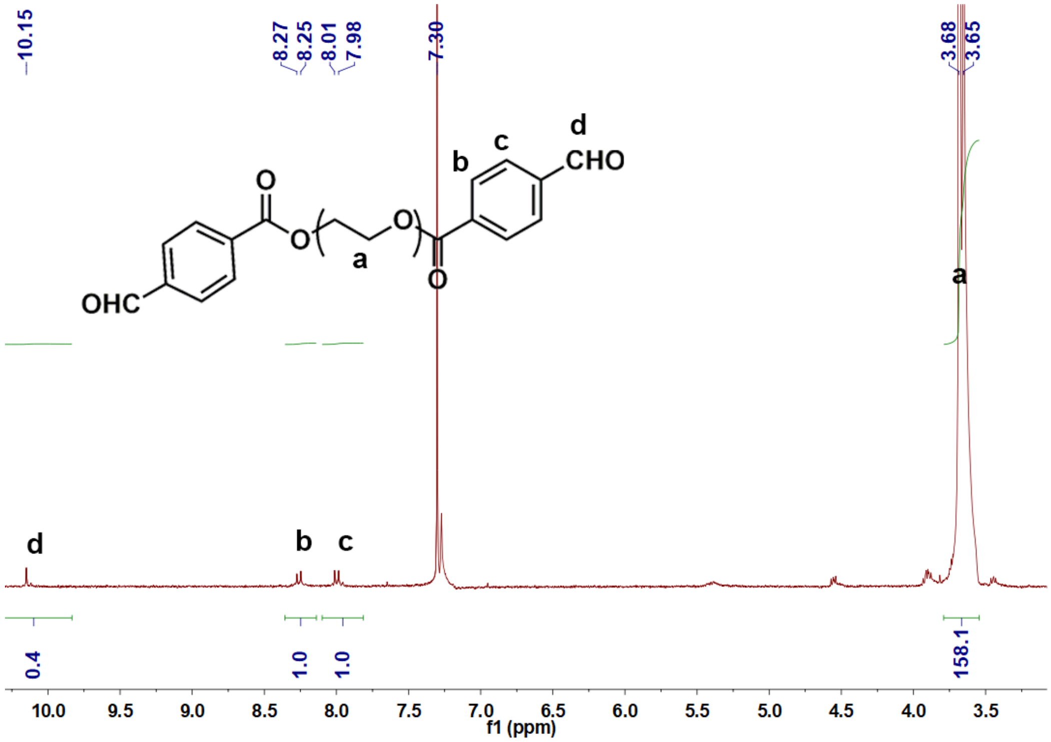

Supplementary Fig. 8 ^{1}H NMR of dual aldehyde end groups of PEG. Repeated for three times in independent experiment.

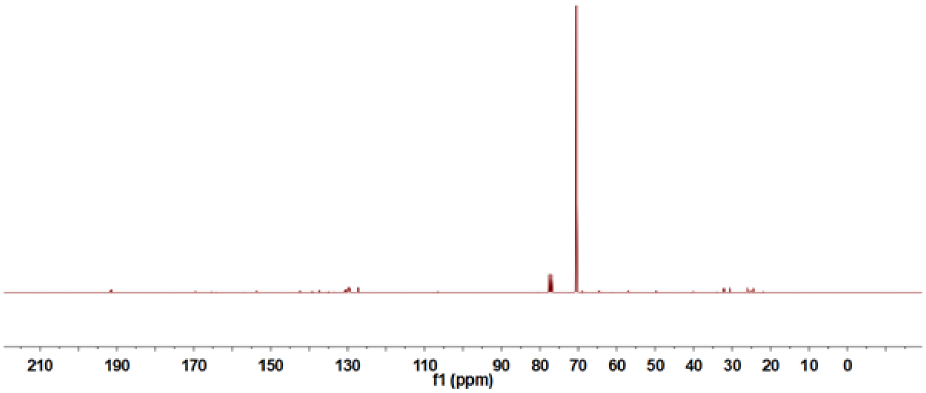

Supplementary Fig. 9 ^{13}C NMR of dual aldehyde end groups of PEG. Repeated for three times in independent experiment.

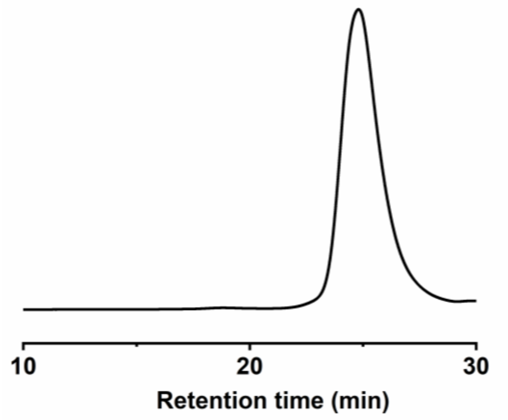

Supplementary Fig. 10 GPC of dual aldehyde end groups of PEG. Repeated for three times in independent experiment.

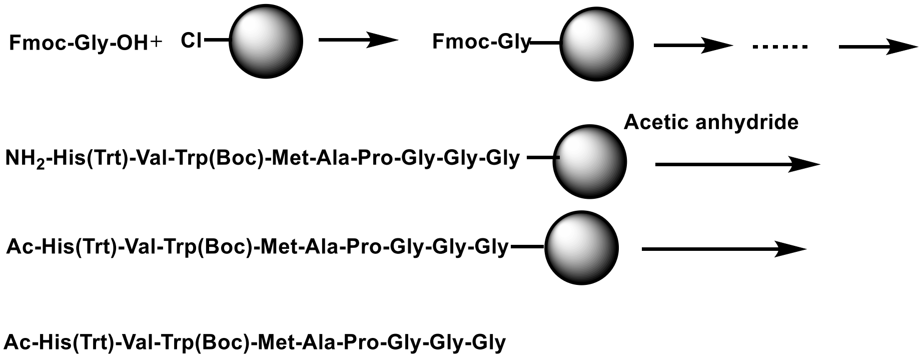

Supplementary Fig. 11 Synthesis route of collagen targeting peptides (T^{ECM}). Repeated for three times in independent experiment.

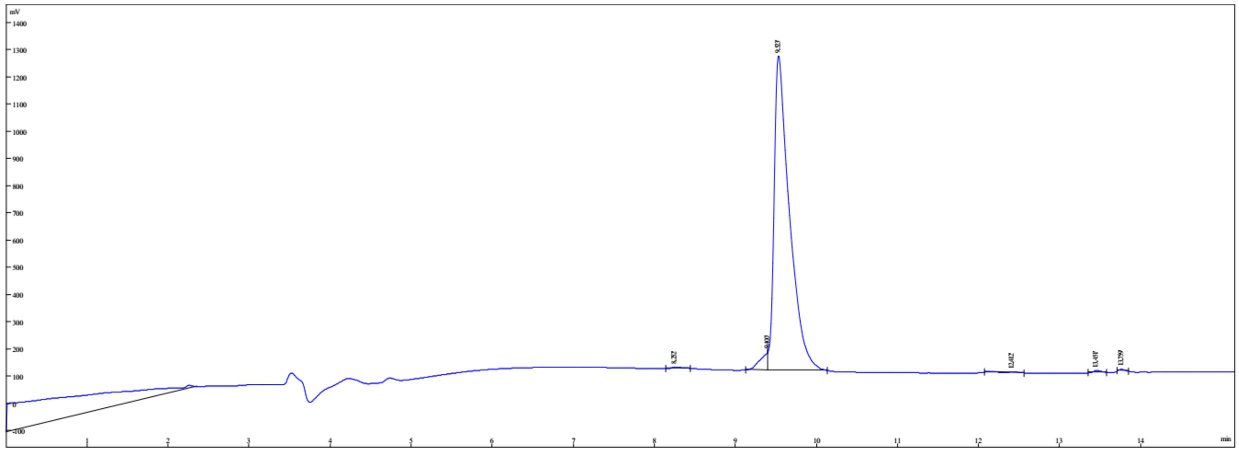

Supplementary Fig. 12 HPLC of collagen targeting peptides (T^{ECM}). Repeated for three times in independent experiment.

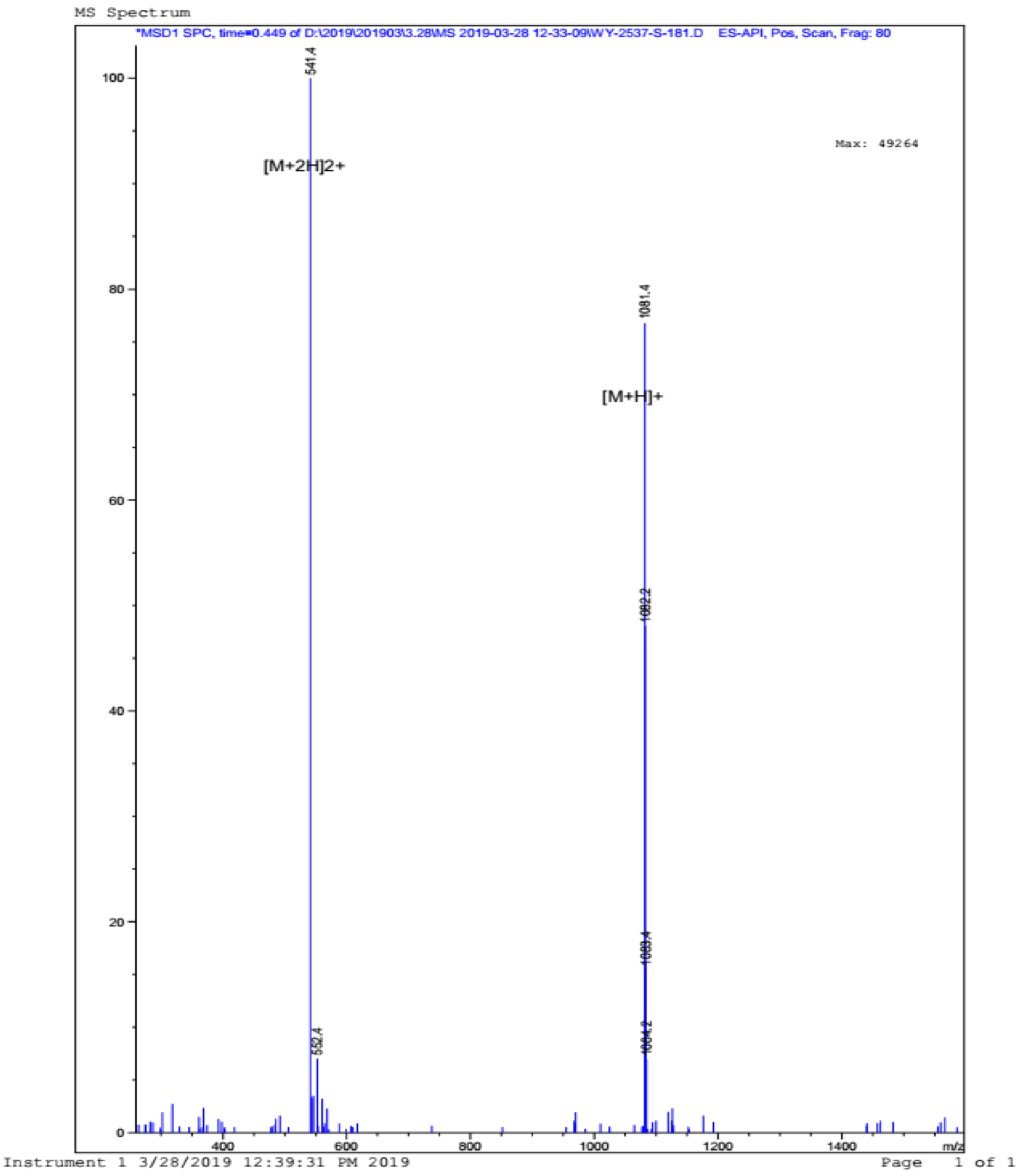

Supplementary Fig. 13 LC-MS of collagen targeting peptides (T^{ECM}). Repeated for three times in independent experiment.

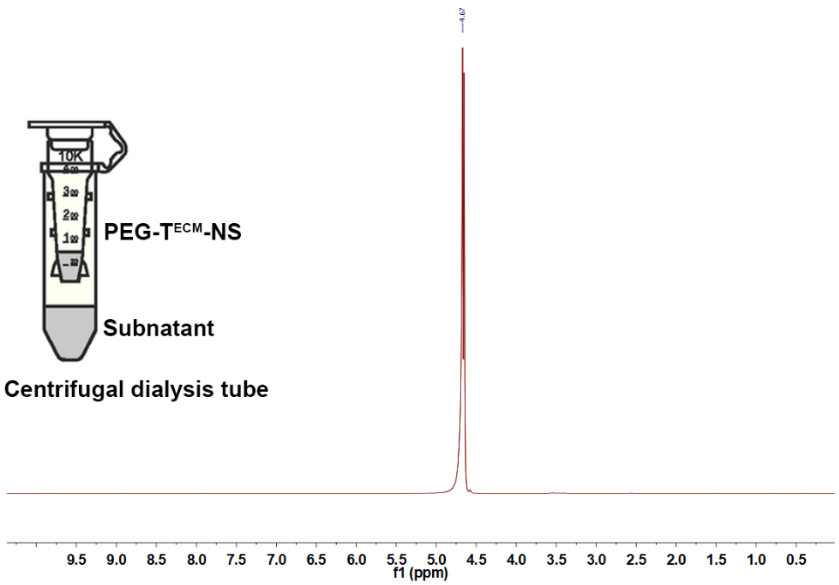

Supplementary Fig. 14 After coating PEG on the surface of T^{ECM}-NS, PEG- T^{ECM}-NS solution was centrifuged using centrifugal dialysis tube and subnatant solution was collected. We use the centrigugal dialysis tube of molecular weight cut-off (MWCO) 10, 000 Da to remove the free PEG. The amount of free PEG in the subnatant was evaluated by ^{1}H NMR. No peak of PEG on the NMR result indicated that there no free PEG. Repeated for three times in independent experiment

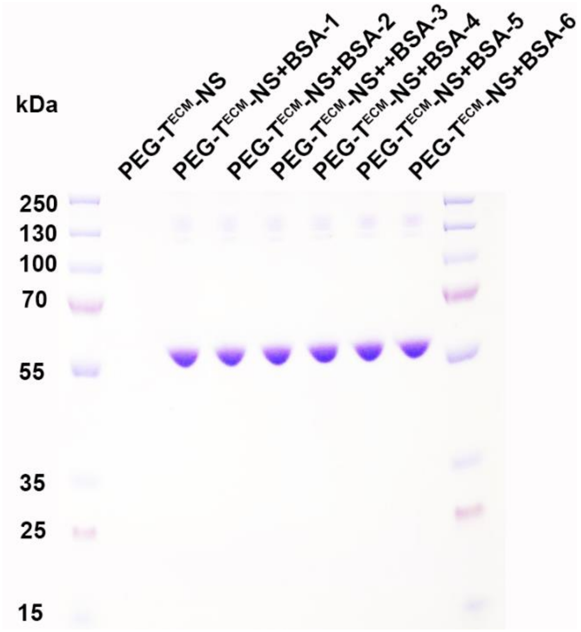

Supplementary Fig. 15 The ability of BSA adsorption on PEG-T^{ECM}-NS was investigated by native PAGE; PEG-T^{ECM}-NS+BSA-1, PEG-T^{ECM}-NS+BSA-2, PEG-T^{ECM}-NS+BSA-3, PEG-T^{ECM}-NS+BSA-4, PEG-T^{ECM}-NS+BSA-5 and PEG-T^{ECM}-NS+BSA-6 represent PEG-T^{ECM}-NS incubated with BSA for 1, 2, 3, 4, 5, and 6 h, respectively, at 37 °C. Repeated for three times in independent experiment.

Supplementary Table 1 Charateristics of OLE loaded PEG-T^{ECM}-NS

	DLC (%)	DLE (%)	Size (nm)	PDI
PEG-T^{ECM}-NS	1.45	29.6	101$\pm$12	0.23$\pm$0.05

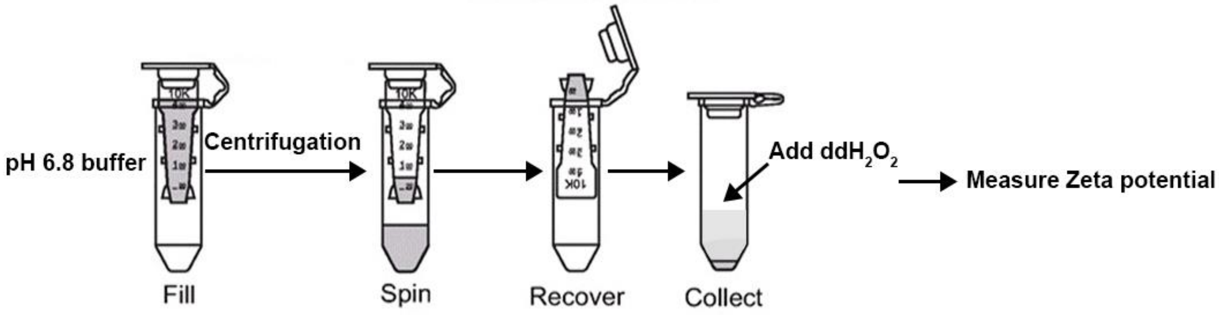

Supplementary Fig. 16 Illustration of de-shielding PEG form PEG-T^{ECM}-NS.

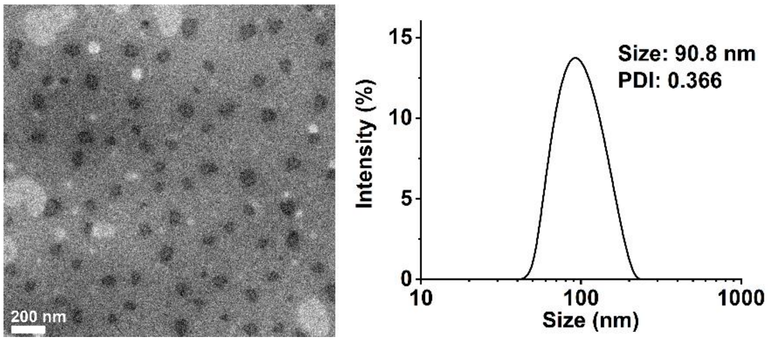

Supplementary Fig. 17 Size distribution of nanoscavenger after de-shielding PEG. Repeated for three times in independent experiment.

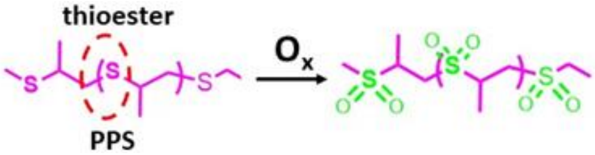

Supplementary Fig. 18 Schematic representation of therapeutic NPs scavenging tumor microenvironment ROS.

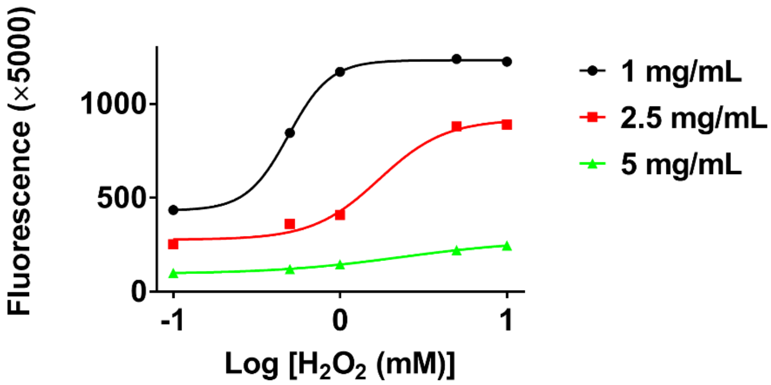

Supplementary Fig. 19 A H_2O_2 activity kit was used to monitor the concentration of H_2O_2 in solution treated with or without T^{ECM}-NS. After incubation with T^{ECM}-NS at different concentration, the significant decrease in fluorescence indicated that the consumption of H_2O_2 by T^{ECM}-NS. Repeated for three times in independent experiment.

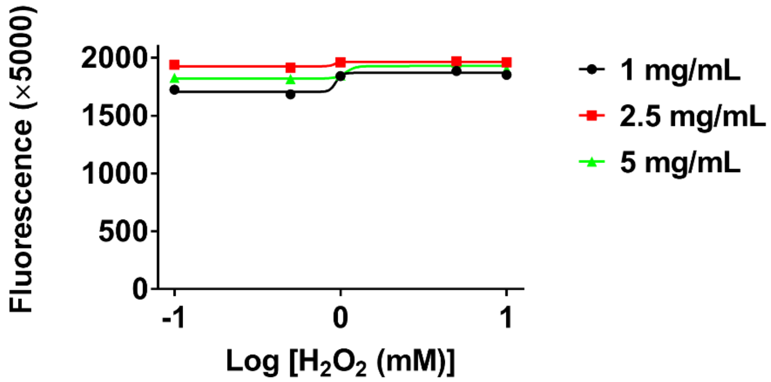

Supplementary Fig. 20 A H_2O_2 activity kit was used to monitor the concentration of H_2O_2 in solution treated with or without PEG-T^{ECM}-C_{18}. Repeated for three times in independent experiment.

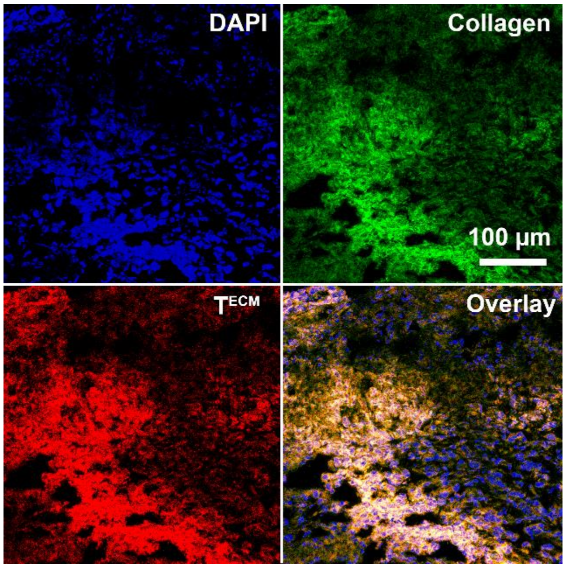

Supplementary Fig. 21 CLSM images of the slices sectioned from the 4T1 tumour pieces (1 cm^3) treated with free collagen targeting peptides (T^{ECM}) for about 4 h. Blue channel, nucleus; green channel, collagen and red channel, RB-labelled T^{ECM}. Repeated for three times in independent experiment.

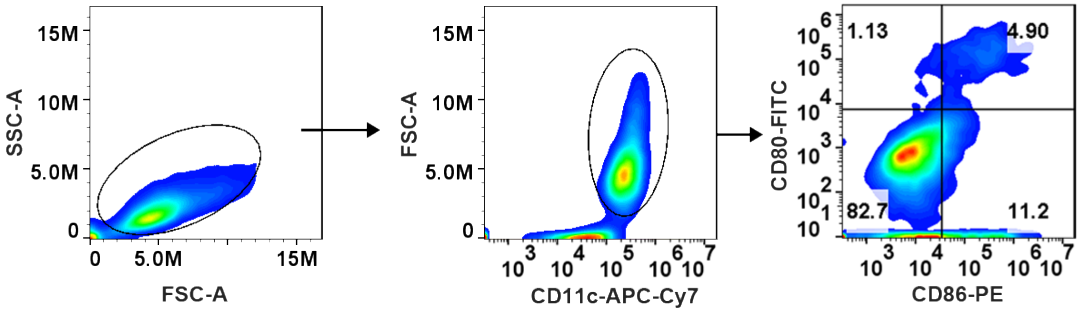

Supplementary Fig. 22 Representative flow cytometry gating strategies for $CD11c^+CD80^+CD86^+$ BMDCs. Repeated for three times in independent experiment.

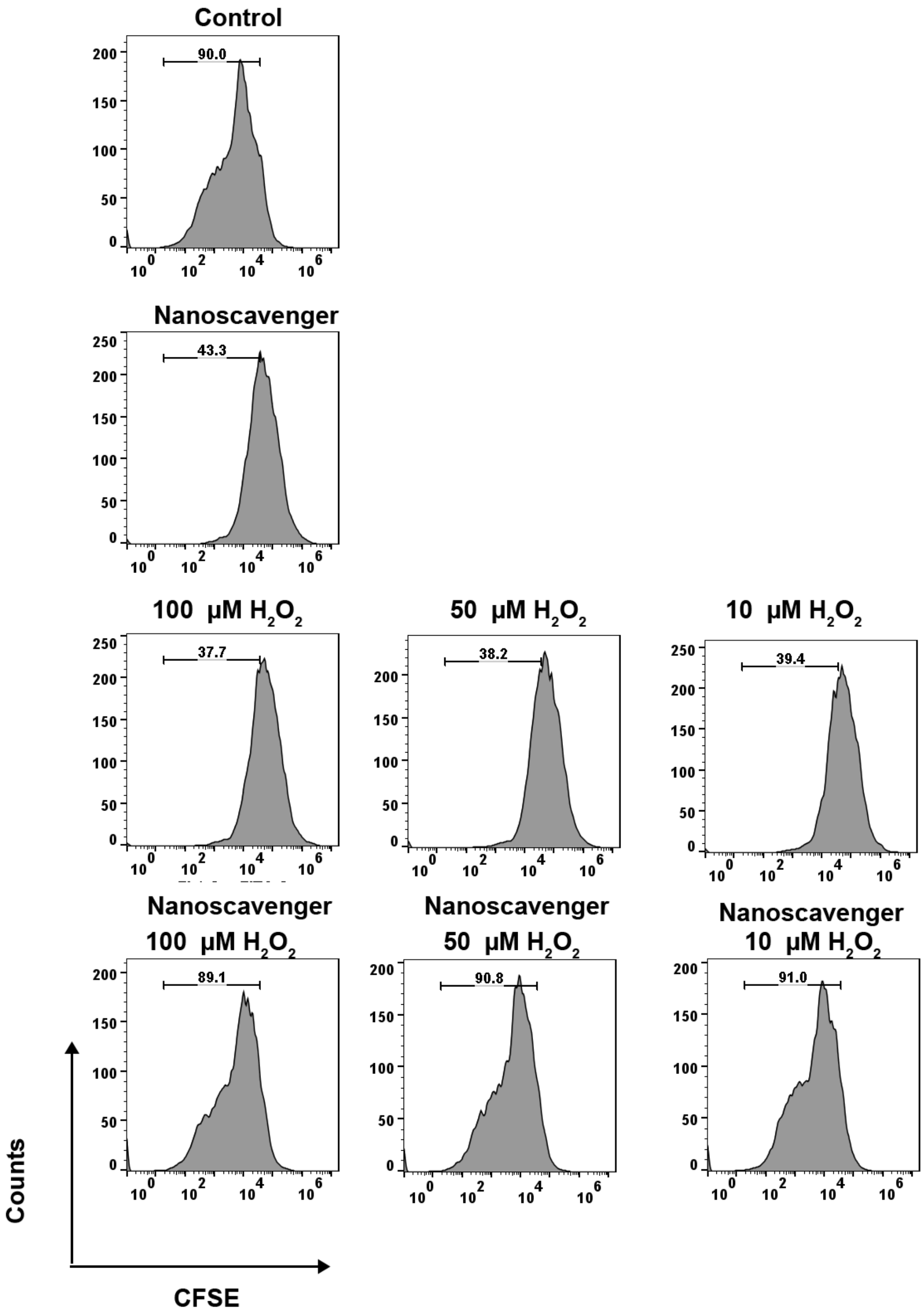

Supplementary Fig. 23 T cell proliferation after different concentration H_2O_2 treatment. Repeated for three times in independent experiment

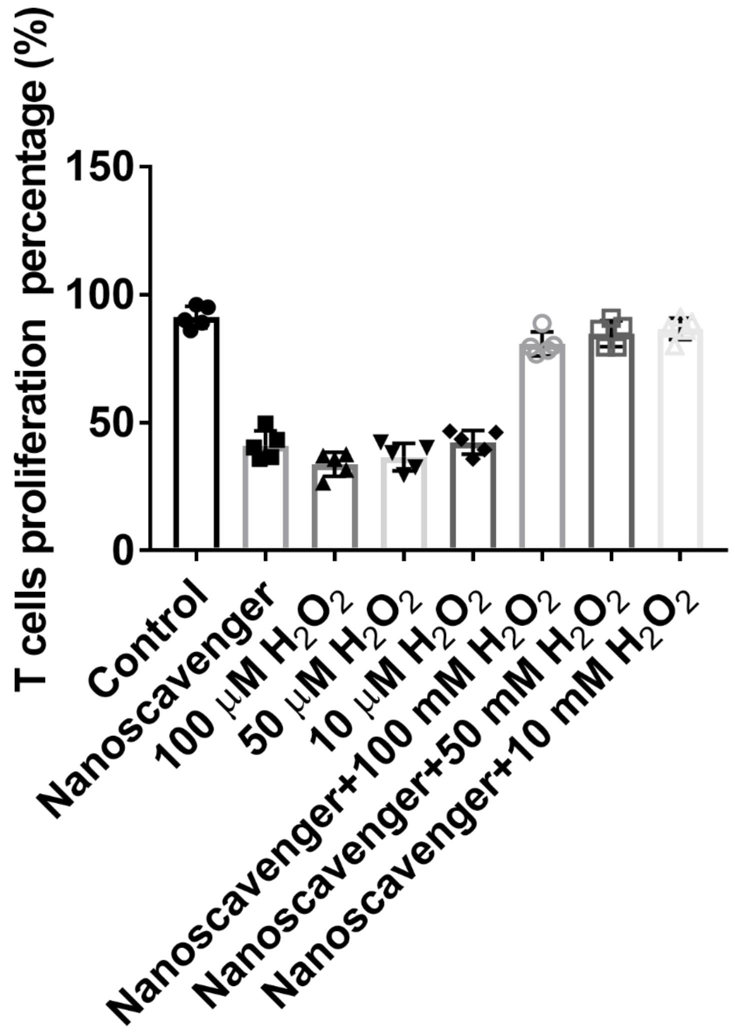

Supplementary Fig. 24 Quantification of T cell proliferation percentage (n = 5 independent replicates). All error bars represent mean ± s.d..

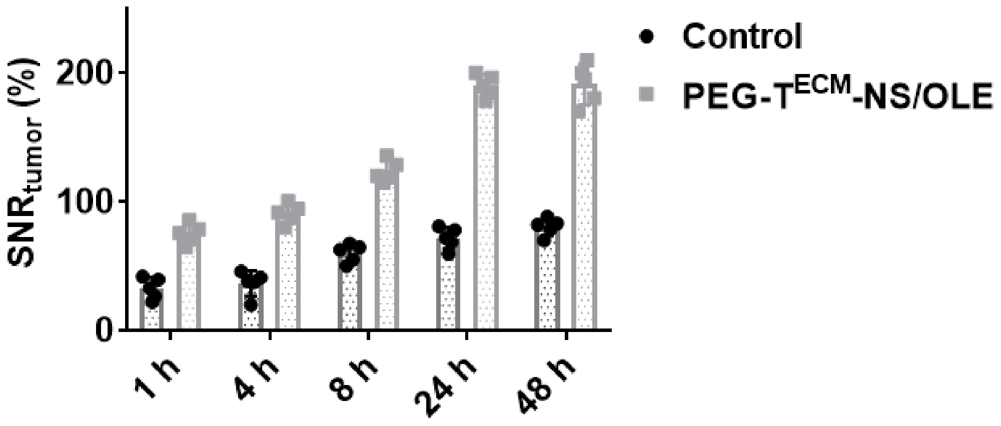

Supplementary Fig. 25 Semi-quantitative analysis of the signal-to-noise ratio (SNR) in tumors (n = 5 biologically independent mice). All error bars represent mean ± s.d..

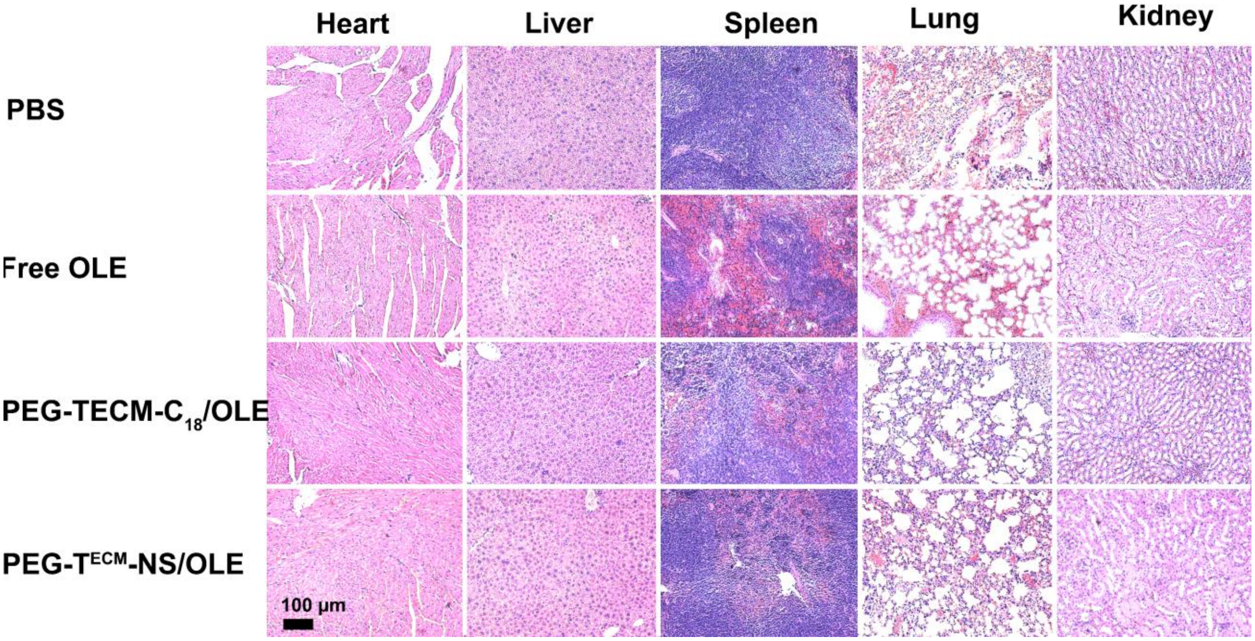

Supplementary Fig. 26 Representative H&E-stained major organ tissues sections from BALB/C mice. Repeated for three times in independent experiment.

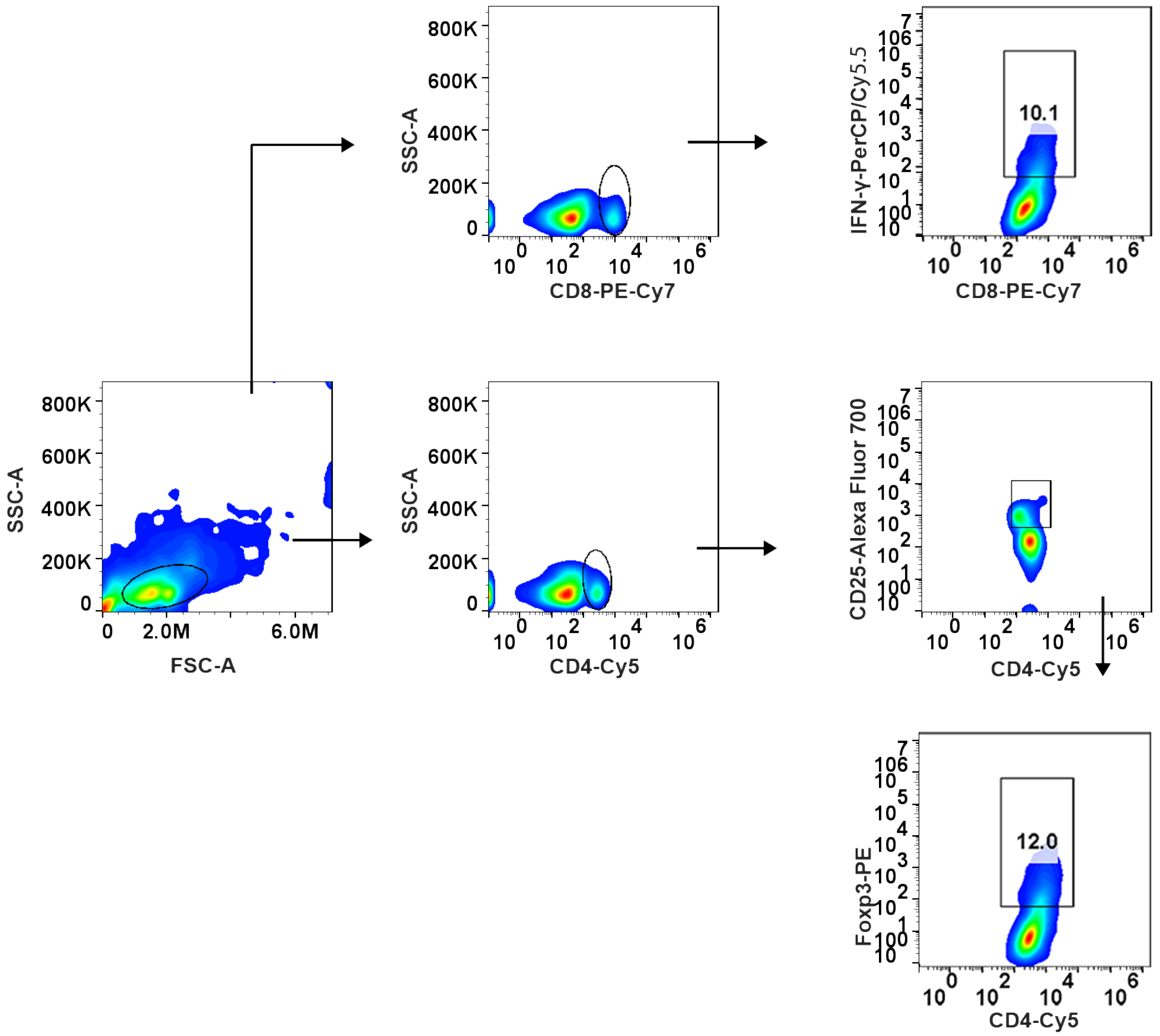

Supplementary Fig. 27 Representative flow cytometry gating strategies for $CD4^{+}Foxp3^{+}$ Tregs and CD8+IFN-γ^{+} T cells. Repeated for three times in independent experiment.
